# Sequelae due to bacterial meningitis among African children: a systematic literature review

**DOI:** 10.1186/1741-7015-7-47

**Published:** 2009-09-14

**Authors:** Meenakshi Ramakrishnan, Aaron J Ulland, Laura C Steinhardt, Jennifer C Moïsi, Fred Were, Orin S Levine

**Affiliations:** 1 2123 Cypress Street, Philadelphia, PA, USA; 2 Sabin Vaccine Institute, Washington, DC, USA; 3 Department of International Health, Johns Hopkins Bloomberg School of Public Health, Baltimore, MD, USA; 4 Kenya Paediatric Association, Nairobi, Kenya

## Abstract

**Background:**

African children have some of the highest rates of bacterial meningitis in the world. Bacterial meningitis in Africa is associated with high case fatality and frequent neuropsychological sequelae. The objective of this study is to present a comprehensive review of data on bacterial meningitis sequelae in children from the African continent.

**Methods:**

We conducted a systematic literature search to identify studies from Africa focusing on children aged between 1 month to 15 years with laboratory-confirmed bacterial meningitis. We extracted data on neuropsychological sequelae (hearing loss, vision loss, cognitive delay, speech/language disorder, behavioural problems, motor delay/impairment, and seizures) and mortality, by pathogen.

**Results:**

A total of 37 articles were included in the final analysis representing 21 African countries and 6,029 children with confirmed meningitis. In these studies, nearly one fifth of bacterial meningitis survivors experienced in-hospital sequelae (median = 18%, interquartile range (IQR) = 13% to 27%). About a quarter of children surviving pneumococcal meningitis and *Haemophilus influenzae *type b (Hib) meningitis had neuropsychological sequelae by the time of hospital discharge, a risk higher than in meningococcal meningitis cases (median = 7%). The highest in-hospital case fatality ratios observed were for pneumococcal meningitis (median = 35%) and Hib meningitis (median = 25%) compared to meningococcal meningitis (median = 4%). The 10 post-discharge studies of children surviving bacterial meningitis were of varying quality. In these studies, 10% of children followed-up post discharge died (range = 0% to 18%) and a quarter of survivors had neuropsychological sequelae (range = 3% to 47%) during an average follow-up period of 3 to 60 months.

**Conclusion:**

Bacterial meningitis in Africa is associated with high mortality and risk of neuropsychological sequelae. Pneumococcal and Hib meningitis kill approximately one third of affected children and cause clinically evident sequelae in a quarter of survivors prior to hospital discharge. The three leading causes of bacterial meningitis are vaccine preventable, and routine use of conjugate vaccines could provide substantial health and economic benefits through the prevention of childhood meningitis cases, deaths and disability.

## Background

Bacterial meningitis is a serious, often disabling and potentially fatal infection resulting in 170,000 deaths worldwide each year [1]. Young children are particularly vulnerable to bacterial meningitis, and when exposed poor outcomes may occur due to the immaturity of their immune systems. Two thirds of meningitis deaths in low-income countries occur among children under 15 years of age [2]. The main bacterial pathogens causing meningitis beyond the neonatal period are *Streptococcus pneumoniae *(pneumococcus), *Haemophilus influenzae *type b (Hib) and *Neisseria meningitidis *(meningococcus) [3-5]. Pneumococcal meningitis is associated with the highest case fatality ratios (CFRs) globally [6]. In Africa, pneumococcal meningitis CFRs attain 45% compared to 29% for Hib meningitis and 8% for meningococcal meningitis [3].

Serious, long-term neuropsychological sequelae further increase the population impact of paediatric meningitis. Sequelae comprise a range of findings with implications for child development and functioning and include such deficits as hearing loss, vision loss, cognitive delay, speech/language disorder, behavioural problems, motor delay/impairment, and seizures [7,8]. Meningitis sequelae can present a long-term, serious hardship for families with limited means to care for a disabled child, especially in resource-poor settings.

Africa experiences a disproportionately large burden of meningitis due to its young population, epidemics in the meningitis belt and high rates of endemic disease. The incidence and CFRs associated with paediatric Hib and pneumococcal meningitis were highest in Africa compared to all other regions in a recent global review [9]. In addition, Africa is the only region with cyclic epidemics of meningitis that affect persons of all ages, with attack rates ranging from 100 to 800 per 100,000 population [10]. Epidemics of meningitis are mostly associated with meningococcus, but there is some evidence that increases in pneumococcal meningitis cases occur in parallel during the hot and dry season [11-13].

A safe and effective Hib conjugate vaccine is available and routinely used in most African countries. Pneumococcal vaccines are gradually being introduced with support from the Global Alliance for Vaccines and Immunisation (GAVI), and meningococcal vaccines will soon become available. Together with mortality, morbidity due to in-hospital and long-term neuropsychological sequelae must be incorporated into estimates of the burden of meningitis to evaluate the potential benefit of preventive efforts such as conjugate vaccines.

The objective of this paper is to present a systematic review of the sequelae following acute bacterial meningitis in African children between the ages of 1 month and 15 years. Several earlier reviews have examined mortality associated with bacterial meningitis [3,9]. This review focuses on studies with primary data on neuropsychological sequelae due to bacterial meningitis.

## Methods

### Literature search strategy

We conducted a systematic literature search for articles published between January 1980 and August 2008 containing information on bacterial meningitis among children from continental Africa. Professional librarians searched eight databases: PubMed/Medline, Global Health Database, Embase, Biological Abstracts, Pascal, Current Contents, the Cochrane Library, and African Index Medicus. Search terms were based on the key words 'meningitis' and at least one of the following: bacteria, bacteraemia, *Streptococcus pneumoniae*, *Haemophilus influenzae *type b, or *Neisseria meningitidis *(see Additional file 1 for full search strategy). We also screened the reference lists of two global reviews for articles with published or unpublished data [5,9]. Citations were uploaded into an EndNote XI library (EndNote, Carlsbad, CA, USA) and de-duplicated.

### Citation screening

Two screeners reviewed each citation by preset inclusion and exclusion criteria; discrepancies were resolved through a weekly conference call. We only included articles with original sequelae data on at least 30 paediatric cases of laboratory-confirmed bacterial meningitis from 1 or more African countries. Laboratory-confirmed bacterial meningitis was defined as bacterial identification in cerebrospinal fluid (CSF) by culture, Gram stain, rapid antigen test (such as the Binax NOW test (Binax Inc., Scarborough, ME, USA)), latex agglutination or polymerase chain reaction, or bacterial isolation from blood culture accompanied by CSF abnormalities such as high white blood cell count, low glucose or high protein. We accepted the article definitions of CSF abnormalities, including CSF white blood cell count greater than 10 to 100 cells/mm^3^, protein greater than 0.3 to 2 g/l, and CSF glucose less than 0.2 g/l or CSF glucose: blood glucose ratio lower than 0.5. Articles had to present sequelae data separately for children or have a majority of their subjects between the ages of 1 month and 15 years. Articles in all languages were considered for inclusion.

### Data extraction

Detailed information on neuropsychological sequelae was collected and entered into a Microsoft Access database (Microsoft, Redmond, WA, USA). Sequelae included hearing loss, vision loss, cognitive delay (including mental retardation and learning disability), speech/language disorder, behavioural problems, motor delay/impairment (including gross motor and fine motor impairment, impaired activities of daily living, hypertonia, and paralysis), seizures, and other neurological sequelae. In-hospital sequelae and deaths were defined as events that occurred after the initiation of treatment and prior to discharge, exclusive of complications observed at the time of admission. Post-discharge sequelae and deaths were defined as events that occurred or persisted after discharge as detected upon follow-up tracing of patients. Unless there was specific mention of patient follow-up after discharge, we assumed that sequelae were assessed in hospital.

We examined articles for overlaps in study population and outcomes: when these occurred, one article in the overlapping group was excluded or the outcomes partially extracted to avoid double counting cases. We also evaluated the consistency of papers by verifying that the numbers presented in the results section added up to the total subjects and total meningitis cases. If there were major or numerous inconsistencies in a paper, it was excluded from the final analysis.

### Data analysis

The final database was transferred into Stata 10.0 for analysis (Stata, College Station, TX, USA). Outcomes of interest included risk of sequelae among survivors (both in hospital and post discharge) and case fatality ratios (in hospital and post discharge). Sequelae and mortality were calculated separately for pneumococcal, Hib, and meningococcal meningitis, and then for all laboratory-confirmed bacterial meningitis. For in-hospital sequelae prevalence, the denominator included all children surviving until discharge and evaluated for sequelae; for post-discharge sequelae prevalence, the denominator included children surviving until the follow-up visit and evaluated for sequelae. Case fatality ratios were calculated only for articles with at least 25 cases of confirmed bacterial meningitis and sequelae risks were calculated only for articles that assessed at least 25 survivors of bacterial meningitis.

African countries were grouped into regions to match the Global Burden of Disease analysis [9]: eastern Africa meningitis belt, middle Africa meningitis belt, western Africa meningitis belt, eastern Africa, middle Africa, western Africa, northern Africa and southern Africa (Table 1).

**Table 1:** List of countries by African region

**Region**	**Countries**
Africa Eastern Meningitis Endemic	Eritrea, Ethiopia, Kenya, Sudan, Uganda
Africa Middle Meningitis Endemic	Cameroon, Central African Republic, Chad
Africa Western Meningitis Endemic	The Gambia, Ghana, Senegal, Togo, Benin, Burkina Faso, Cote d'Ivoire, Guinea, Guinea-Bissau, Mali, Mauritania, Niger, Nigeria
Africa Eastern	Mauritius, Seychelles, Comoros, Djibouti, Madagascar, Zimbabwe, Burundi, Malawi, Mozambique, Rwanda, Somalia, United Republic of Tanzania, Zambia
Africa Middle	Congo, Gabon, Angola, Democratic Republic of Congo, Equatorial Guinea
Africa Western	Cape Verde, Sao Tome and Principe, Liberia, Sierra Leone
Africa Northern	Libya, Tunisia, Algeria, Egypt, Morocco
Africa Southern	Namibia, South Africa, Botswana, Lesotho, Swaziland

African countries are grouped into eight regions, based on the groupings used in the Global Burden of Disease analysis [9].

## Results

### Literature search and citation screening

**Figure 1:**
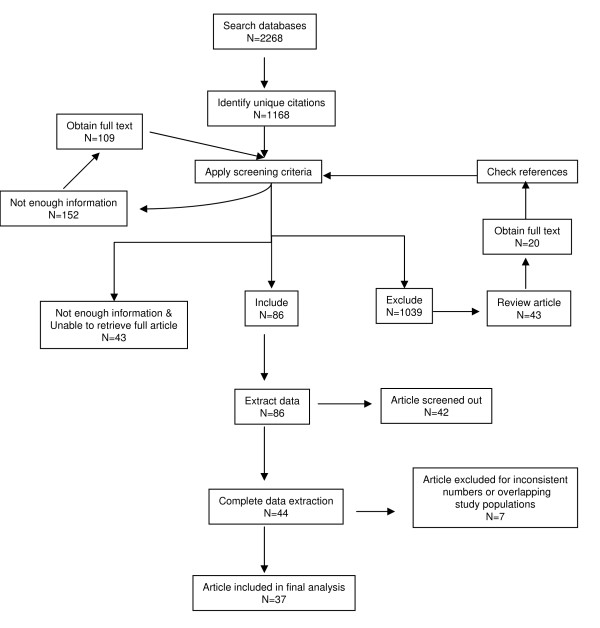
**Citations found through literature search**.

We initially identified 2,268 citations, of which 1,168 remained after de-duplication (Figure 1). All unique citations were double screened and 1,039 were excluded. Among these, 43 review articles were identified of which 20 were retrieved in full and checked for pertinent, unique citations; no new citations were found through this process. A total of 43 articles could not be retrieved prior to 1 October 2008 and were hence excluded.

In all, 86 articles were moved forward to the data extraction phase. Of those articles, 42 were excluded because they did not present sequelae data separately for laboratory-confirmed and unconfirmed cases of bacterial meningitis. An additional 7 articles were excluded due to inconsistencies in the data presented in the results; thus, 37 articles remained in the final analysis.

### Articles included in the review

**Figure 2:**
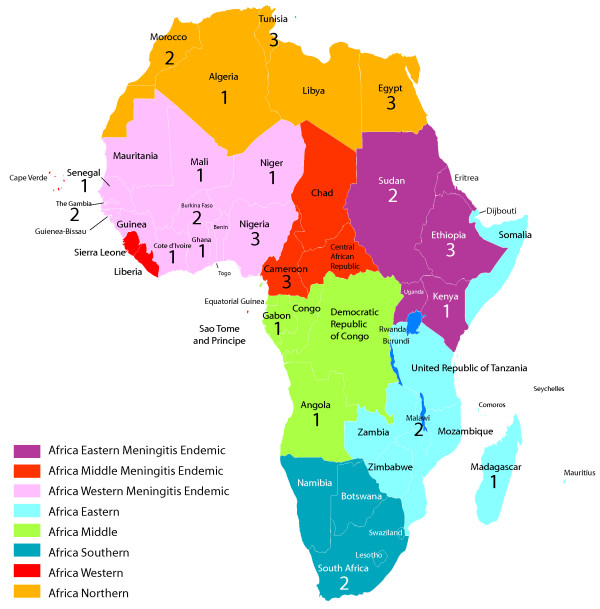
**Number of articles included in review by country and region**.

The 37 included articles present data from 21 different African countries (Figure 2 and Table 2). A total of 10 studies were conducted during a meningitis epidemic. All regions contributed some data on in-hospital sequelae. The western Africa meningitis belt region had the most data, with 12 articles including 2,394 cases of confirmed bacterial meningitis (CBM) with known outcomes, while southern Africa had the least, with 2 articles including 140 cases of CBM with known outcomes (Table 3). Post-discharge sequelae data were more limited, and originated from only seven countries in northern Africa or the meningitis belt. Only 1,131 children with any type of CBM were assessed for post-discharge sequelae.

**Table 2:** List of articles included in review

**Reference**	**First author**	**Year of publication**	**Country**	**Region**	**Total no. of CBM cases**
[33]	Abid	1999	Morocco	Africa Northern	141
[34]	Abid	1999	Morocco	Africa Northern	118
[35]	Akpede	1999	Nigeria	Western Meningitis	63
[36]	Bijlmer	1990	Gambia	Western Meningitis	77
[37]	Bissagnene	1996	Cote d'Ivoire	Western Meningitis	654
[38]	Bernard-Bonnin	1985	Cameroon	Middle Meningitis	174
[39,40]	Camara	2003	Senegal	Western Meningitis	511
[14]	Friedland	1992	South Africa	Africa Southern	79
[16]	Girgis	1991	Egypt	Africa Northern	160
[41]	Girgis	1989	Egypt	Africa Northern	429
[17]	Girgis	1998	Egypt	Africa Northern	697
[22]	Goetghebuer	2000	The Gambia	Western Meningitis	257
[42]	Grobler	1997	South Africa	Africa Southern	61
[43]	Hailu	2001	Ethiopia	Eastern Meningitis	32
[44]	Koko	2000	Gabon	Africa Middle	91
[45]	Kristos	1993	Ethiopia	Eastern Meningitis	124
[46]	Lagunju		Nigeria	Western Meningitis	29
[47]	Mackie	1992	Ghana	Western Meningitis	69
[48]	Mbonda	1995	Cameroon	Middle Meningitis	67
[49]	Melaku	2003	Ethiopia	Eastern Meningitis	53
[50]	Mhirsi	1992	Tunisia	Africa Northern	98
[51]	Molyneux	1998	Malawi	Africa Eastern	149
[52]	Molyneux	2000	Malawi	Africa Eastern	52
[53]	Moreau	1986	Cote d'Ivoire	Western Meningitis	47
[54]	Mwangi	2002	Kenya	Eastern Meningitis	224
[55]	Ogunlesi	2005	Nigeria	Western Meningitis	124
[56]	Pelkonen	2008	Angola	Africa Middle	403
[57,58]	Razafindralambo	2004	Madagascar	Africa Eastern	83
[59]	Redjah	1998	Algeria	Africa Northern	57
[60,61]	Salih	1990	Sudan	Eastern Meningitis	43
[62]	Salih	1990	Sudan	Eastern Meningitis	108
[63]	Sangare	2005	Burkina Faso	Western Meningitis	53
[64]	Mefo	1999	Cameroon	Middle Meningitis	99
[65]	Soltani	2005	Tunisia	Africa Northern	20
[66]	Tall	1992	Burkina Faso	Western Meningitis	285
[15]	Thabet	2007	Tunisia	Africa Northern	73
[67]	WHO	1993	Mali and Niger	Western Meningitis	426

The 37 articles included in this review are listed in the above table. Articles with overlapping data have two references listed in the 'reference' column but were extracted as one article in the review database.

CBM = confirmed bacterial meningitis; WHO = World Health Organization.

**Table 3:** Numbers of articles and cases reviewed by region

**Region**	**No. articles**	**Total no. confirmed BM cases with follow-up**	**Median no. confirmed BM cases with follow-up (min, max)**	**Total no. evaluated for in-hospital sequelae**	**Total no. evaluated for post-discharge sequelae**
Eastern Meningitis	6	584	81 (32, 224)	431	80
Middle Meningitis	3	340	99 (67, 174)	211	67
Western Meningitis	12	2,394	85 (23, 654)	1,353	168
Africa Eastern	3	284	83 (52, 149)	173	0
Africa Middle	2	494	(91, 403)	332	0
Africa Northern	9	1,793	118 (20, 697)	457	816
Africa Southern	2	140	(61, 79)	104	0
Total	37	6,029		3,061	1,131

A total of 3,061 survivors were evaluated for in-hospital sequelae. Most of the in-hospital sequelae data comes from the western Africa meningitis belt region. Much less data is available on post-discharge sequelae (n = 1,131 cases) because survivors of bacterial meningitis were lost to follow-up or died after hospital discharge. Most of the post-discharge data comes from the northern Africa region.

BM = bacterial meningitis.

### In-hospital sequelae and mortality

#### All causes of CBM

In all, 27 articles reported in-hospital mortality data for 25 or more children with CBM (4,245 total cases); 24 of these articles also had sequelae data for at least 25 child survivors (2,971 total cases) (Table 4). Most studies (23 of 37, or 62%) included pneumococcus, Hib, and/or meningococcus. Nine studies also included other bacterial pathogens such as *Salmonella*, enterobacteria, *Staphylococcus*, other streptococci, *Escherichia coli *or *Klebsiella*. One study included only tuberculosis (TB) meningitis cases.

**Table 4:** In-hospital outcomes for all causes of confirmed bacterial meningitis (CBM), by study

**Reference**	**Country**	**No. CBM cases with known outcomes**	**CFR**	**No. cases assessed for sequelae**	**HL (%)**	**VL (%)**	**CD (%)**	**BP (%)**	**MI (%)**	**SZ (%)**	**Any neuropsychological sequelae (%)**	**Bacterial pathogens studied**
[43]	Ethiopia	32	34%									Spn, Hib, Nm, Gram negative enterococci
[45]	Ethiopia	124	2%	121	3%						8%	Nm
[54]	Kenya	224	33%	150							27%	Spn, Hib, non-typhi *Salmonella*, enterobacteria, *Streptococcus *group A or B and others
[62]	Sudan	108	4%	104	3%				2%		5%	Nm
[60]	Sudan	43	19%	35	20%				11%^a^		26%	Spn, Hib, Nm and others
[38]	Cameroon	174	30%	121							17%	Spn, Hib, Nm, *Streptococcus*, *Staphylococcus*, enterobacteria
[64]	Cameroon	99	9%	90					1%^a^	1%	4%	Spn, Hib, Nm
[63]	Burkina Faso	53	75%									*Salmonella*
[66]	Burkina Faso	92	22%	72					7%		18%	Hib
[37]	Cote d'Ivoire	654	41%	380	6%	1%			2%		19%	Spn, Hib
[53]	Cote d'Ivoire	47	4%	45							7%	Spn, Hib, Nm
[47]	Ghana	67	27%	49	8%	2%			6%^a^		12%	Spn, Hib, Nm
[67]	Mali and Niger	426	37%	269							14%	Spn, Hib, Nm and others
[55]	Nigeria	124	27%	91							18%	Spn, Hib, Nm and others
[40]	Senegal	511	18%	420							21%	Spn, Hib, Nm
[56]	Angola	403	33%	270							24%	Spn, Hib, Nm, others
[57]	Madagascar	83	31%	57							30%	Spn, Hib, Nm
[51]	Malawi	149	34%	92							20%	Spn, Hib, *Escherichia coli*, *Klebsiella*, Group B Streptococcus, others
[52]	Malawi	52	54%									Salmonella
[44]	Gabon	91	31%	62							18%	Spn, Hib, Nm, *Salmonella*
[59]	Algeria	57	4%	55	2%		2%				4%	Hib
[16]	Egypt	160	51%	78		8%			6%		18%	Tuberculosis
[34]	Morocco	118	1%	117	9%	3%		1%	2%	1%	21%	Nm
[33]	Morocco	141	9%	128							27%	Spn
[15]	Tunisia	73	14%	61	7%	2%			5%	3%	34%	Spn
[42]	South Africa	61	20%	49	33%	8%			37%		57%	Spn, Hib, Nm, others
[14]	South Africa	79	30%	55	5%						38%	Spn

This table presents data extracted on in-hospital sequelae among studies with at least 25 CBM survivors and case fatality ratios (CFRs) among studies with at least 25 CBM cases for all bacterial causes combined. The proportion of meningitis survivors with any kind of neuropsychological sequelae, either one or more deficits, ranged from 4% to 57%. The in-hospital CFR ranged from 1% to 75%. In many studies, the proportion of children affected by specific types of sequelae was not given. Most sequelae were diagnosed by clinical exam only during the course of hospitalisation.

^a^Paralysis.

BP = behavioural problem; CD = cognitive delay; Hib = *Haemophilus influenzae *type b; HL = hearing loss; MI = motor impairment; Nm = *Neisseria meningitidis*; Spn = *Streptococcus pneumoniae*; SZ = seizures; VL = vision loss;

Cases were recruited and assessed for sequelae prospectively in 12 of 24 studies and retrospectively in 11 studies. One study recruited cases retrospectively and assessed outcomes prospectively. In 20 studies, sequelae were diagnosed by clinical exam only.

**Figure 3:**
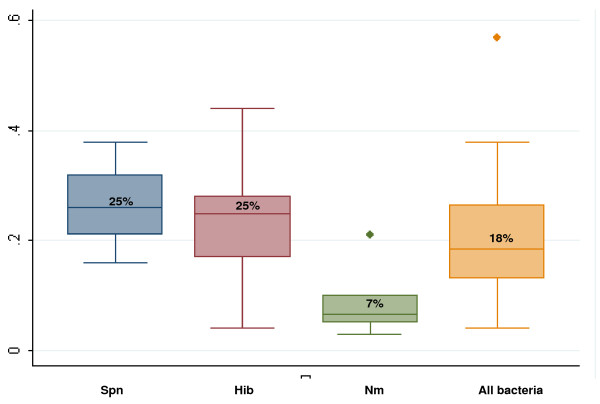
**Box plots of proportion of survivors with in-hospital sequelae**. This figure presents box plots of the range of estimates for any in-hospital neuropsychological sequelae by pathogen and for all causes of confirmed bacterial meningitis (CBM) combined. The upper border of the shaded box is the value of the 75th percentile and the lower border of the shaded box is the 25th percentile, and these two values define the interquartile range (IQR). The vertical 'whiskers' represent the values 1.5 IQRs above the 75th percentile and 1.5 IQRs below the 25th percentile. Any data points that are beyond the whiskers appear as outliers (dots). The median estimate for each group is represented by the horizontal line within the shaded box and the number above the horizontal line. Spn = *Streptococcus pneumoniae*, Hib = *Haemophilus influenzae type b*, Nm = *Neiserria meningitidis*

The risk of in-hospital neuropsychological sequelae (Figure 3) ranged from 4% to 57% for all causes of bacterial meningitis combined (median 18%, interquartile range (IQR) 13% to 27%). A total of 12 studies provided information by sequelae type. Hearing loss and motor impairments were most frequently reported, with estimates of risk ranging from 2% to 33% and 1% to 37%, respectively, based on 10 studies each. No studies had data on speech or language problems.

**Figure 4:**
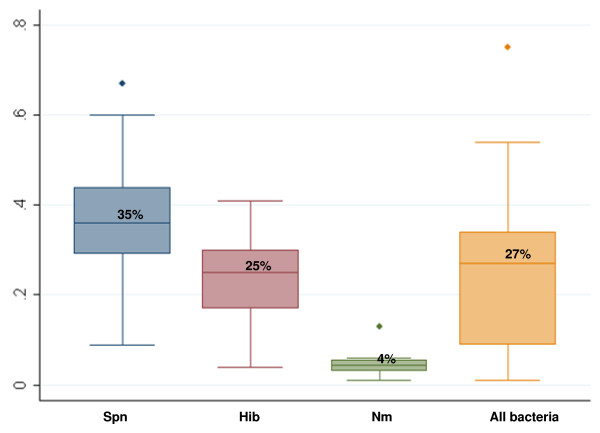
**Box plots of in-hospital case fatality ratios**. Spn = *Streptococcus pneumoniae*, Hib = *Haemophilus influenzae type b*, Nm = *Neiserria meningitidis*

CFRs for all confirmed types of bacterial meningitis ranged from 1% to 75%, (median 27%, IQR 9% to 34%) (Figure 4).

#### Pneumococcal meningitis

A total of 10 studies had data on pneumococcal meningitis sequelae, including 676 children. These studies found one or more sequelae in 16% to 38% of children (median 25%, IQR 21% to 32%) (Table 5). One study found hearing loss in 5% of cases [14], and another study examining hearing loss, vision loss, motor delay and seizures found that 2% to 7% of children had any one of these specific deficits [15]. A total of 14 articles including 1,463 children with pneumococcal meningitis provided information on CFR, which ranged from 9% to 67% (median 35%, IQR 29% to 44%).

**Table 5:** In-hospital outcomes for the three main bacterial pathogens, by study

**Reference**	**Bacteria**	**Country**	**No. of CBM cases**	**In-hospital CFR**	**Total no. cases assessed for sequelae**	**HL (%)**	**VL (%)**	**CD (%)**	**BP (%)**	**MI (%)**	**SZ (%)**	**Any neuropsychological sequelae (%)**
[38]	Spn	Cameroon	74	39%	45							16%
[37]	Spn	Cote d'Ivoire	330	60%	132							25%
[47]	Spn	Ghana	33	36%								
[67]	Spn	Mali and Niger	115	67%	38							21%
[22]	Spn	The Gambia	134	48%								
[57]	Spn	Madagascar	40	40%								
[51]	Spn	Malawi	62	44%	32							25%
[44]	Spn	Gabon	42	29%	30							20%
[33]	Spn	Morocco	141	9%	128							27%
[15]	Spn	Tunisia	73	14%	61	7%	2%			5%	3%	34%
[41]	Spn	Egypt	106	27%								
[14]	Spn	South Africa	79	30%	55	5%						38%
[54]	Spn	Kenya	92	36%	59							32%
[40]	Spn	Senegal	142	32%	96							28%
[54]	Hib	Kenya	77	17%	64							25%
[60]	Hib	Sudan	25	16%								
[38]	Hib	Cameroon	53	25%	40							15%
[37]	Hib	Cote d'Ivoire	314	21%	248	6%	1%			2%		17%
[36]	Hib	The Gambia	77	25%								
[67]	Hib	Mali and Niger	133	36%	85							28%
[40]	Hib	Senegal	216	18%	178	6%	3%				2%	27%
[22]	Hib	The Gambia	123	27%								
[66]	Hib	Burkina Faso	92	22%	72					7%		18%
[57]	Hib	Madagascar	35	29%	25							44%
[51]	Hib	Malawi	44	41%								
[44]	Hib	Gabon	36	31%								
[59]	Hib	Algeria	57	4%	55	2%		2%				4%
[41]	Hib	Egypt	56	30%								
[42]	Hib	South Africa	35	11%	31	26%				32%		32%^a^
[45]	Nm	Ethiopia	124	2%	121	3%						8%
[62]	Nm	Sudan	108	4%	104	3%				2%		5%
[64]	Nm	Cameroon	77	5%	73					1%^b^	1%	3%
[67]	Nm	Mali and Niger	161	13%	140							5%
[40]	Nm	Senegal	153	5%	146							10%
[41]	Nm	Egypt	267	6%								
[34]	Nm	Morocco	118	1%	117	9%	3%		1%	2%	1%	21%
[50]	Nm	Tunisia	57	4%								

This table presents the in-hospital sequelae and case fatality ratio (CFR) data available for 25 or more children diagnosed or surviving meningitis caused by 1 of the 3 main bacterial causes. Sequelae prevalences ranged from 16% to 38% for Spn meningitis, 4% to 44% for Hib meningitis and 3% to 21% for Nm meningitis.

^a^Estimated 'Any neuropsychological sequelae' based on single sequelae type with largest proportion; ^b^paralysis.

BP = behavioural problem; CBM, confirmed bacterial meningitis; CD = cognitive delay; Hib = *Haemophilus influenzae *type b; HL = hearing loss; MI = motor impairment; Nm = *Neisseria meningitidis*; Spn = *Streptococcus pneumoniae*; SZ = seizures; VL = vision loss.

#### Hib meningitis

Altogether, 9 studies had data on Hib meningitis sequelae, including a total of 798 children. In all, 4% to 44% of survivors had neuropsychological sequelae (median 25%, IQR 17% to 28%) (Table 5). The risk of sequelae ranged from 2% to 26% for hearing loss (four studies), 1% to 3% for vision loss (two studies), 2% to 32% for motor delay/impairment (two studies), and 2% for seizures and 2% for cognitive delay (one study each). Among 15 articles including 1,373 children with Hib meningitis, the CFR ranged from 4% to 41% (median 25%, IQR 17% to 36%).

#### Meningococcal meningitis

In all, 6 studies including a total of 701 children had data on meningococcal meningitis sequelae with prevalence estimates ranging from 3% to 21% (median 7%, IQR 5% to 10%) (Table 5). Studies found hearing loss in 3% to 9% of subjects (three studies), vision loss in 3% (one study), behavioural problems in 1% (one study), motor impairment in 1% to 2% (three studies), and seizures in 1% (two studies). Among 8 studies following 1,065 children, the CFR for meningococcal meningitis ranged from 1% to 13% (median 4%, IQR 3% to 6%).

### Post-discharge sequelae and mortality

A total of 10 prospective studies evaluated children for neuropsychological sequelae after discharge with CBM (Table 6). The follow-up period ranged from 2 to 90 months after hospital discharge. Loss to follow-up of patients ranged from 0% to 49% in eight studies and could not be calculated in two studies. Post-discharge mortality was reported in five studies and ranged from 0% to 18% (median 10%). Sequelae were generally assessed by clinical exam alone. One or more sequelae were found in 3% to 47% of survivors (median 25%, IQR 13% to 33%). The two studies enrolling only children with TB meningitis had sequelae estimates (24% and 32%) similar to studies including other bacterial pathogens [16,17]. Details of sequelae and mortality following discharge with pneumococcal, Hib and meningococcal meningitis are shown in Tables 7 and 8.

**Table 6:** Post-discharge (PD) outcomes for all causes of confirmed bacterial meningitis (CBM), by study

**Reference**	**Country**	**No. cases discharged**	**PD CFR**	**Average follow-up time, months**	**Total no. assessed for sequelae**	**HL (%)**	**VL (%)**	**CD (%)**	**SLD (%)**	**BP (%)**	**MI (%)**	**SZ (%)**	**Any neuropsychological sequelae (%)**	**Bacterial pathogens studied**
[50]	Tunisia	85		60	82								13%	Spn, Hib, Nm
[41]	Egypt	367	0%	3	367	2%	0%	0%			1%		3%	Spn, Hib, Nm
[35]	Nigeria	47	0%		47							15%	23%	Spn, Hib, Nm, *Klebsiella *and others
[16]	Egypt	78		(2 to 24)	78		10%				10%		24%	Tuberculosis
[17]	Egypt	289		12	289		8%				7%		32%	Tuberculosis
[49]	Ethiopia				53	34%							34%^a^	Spn, Hib, Nm, others
[36]	The Gambia	58	16%	8	48								13%	Hib
[48]	Cameroon			14	67	13%	3%	4%	4%	2%	3%^b^	7%	25%	Spn, Hib, Nm, others
[60]	Sudan	34	10%	(3 to 48)	27	22%			7%	7%	7%	11%	33%	Spn, Hib, Nm and others
[22]	The Gambia	160	18%	(11 to 90)	73	33%	11%	22%			27%^c^	19%	47%	Spn, Hib

The outcomes of children treated for all causes of CBM are shown for studies with >25 subjects. The number of cases discharged was calculated based on the number of CBM cases included in the study minus the number of children reported who died in-hospital. Post-discharge case fatality ratio (CFR) was calculated as the number of deaths after discharge divided by the number of cases with known follow-up; the denominator does not include cases lost to follow-up. Sequelae prevalence in children followed-up ranged from 3% to 47%. Half of the studies also had information on post-discharge CFR, which ranged from 0% to 18% in the period 3 to 90 months after illness.

^a^Estimated 'Any neuropsychological sequelae' based on single sequelae type with largest proportion; ^b^paralysis; ^c^gross motor impairment.

BP = behavioural problem; CD = cognitive delay; Hib = *Haemophilus influenzae *type b; HL = hearing loss; MI = motor impairment; Nm = *Neisseria meningitidis*; Spn = *Streptococcus pneumoniae*; SLD = speech or language disorder; SZ = seizures; VL = vision loss.

**Table 7:** Post-discharge outcomes for the three main bacterial pathogens, by study

**Reference**	**Bacteria**	**Country**	**No. cases discharged**	**PD CFR**	**Average follow-up time, months (or range)**	**Total no. assessed for sequelae**	**HL (%)**	**VL (%)**	**CD (%)**	**SLD (%)**	**BP (%)**	**MI (%)**	**SZ (%)**	**Any neuropsychological sequelae (%)**
[41]	Spn	Egypt	77	0%	3	77	5%	0%	0%			0%		5%
[48]	Spn	Cameroon			14	34	21%	6%	9%	9%	3%	6%^a^	9%	41%
[22]	Spn	The Gambia	70	23%	(11 to 90)	31	48%	23%	32%			48%^b^	10%	58%
[41]	Hib	Egypt	39	0%	3	39	0%	0%	0%			0%		0%
[36]	Hib	The Gambia	58	16%	8	48								13%
[22]	Hib	The Gambia	90	14%	(11 to 90)	42	21%	2%	14%			12%^b^	26%	38%
[50]	Nm	Tunisia	55		60	55								9%
[41]	Nm	Egypt	251	0%	3	251	2%	0%	0%			1%^a^		3%

This table shows the findings of studies with post-discharge follow-up of at least 25 children following confirmed bacterial meningitis (CBM) caused by 1 of the 3 main bacterial pathogens. The number of cases discharged was calculated based on the number of CBM cases included in the study minus the number of children reported who died in hospital. Post-discharge case fatality ratio (CFR) was calculated as the number of deaths after discharge divided by the number of cases with known follow-up; the denominator does not include cases lost to follow-up.

^a^Paralysis; ^b^gross motor impairment.

BP = behavioural problem; CD = cognitive delay; Hib = *Haemophilus influenzae *type b; HL = hearing loss; MI = motor impairment; Nm = *Neisseria meningitidis*; Spn = *Streptococcus pneumoniae*; SLD = speech or language disorder; SZ = seizures; VL = vision loss.

**Table 8:** Overview of sequelae and mortality by bacterial pathogen and for all-cause confirmed bacterial meningitis (CBM)

**Type of bacteria**	**No. of studies with in-hospital data**	**Total no. cases**	**In-hospital CFR: median (min, max)**	**% With any in-hospital sequelae: median (min, max)**	**No. of studies with post-discharge data**	**Total no. cases discharged**	**% Cases died after discharge: median (min, max)**	**% With any post-discharge sequelae: median (min, max)**
Pneumococcus	14	1,463	35% (9% to 67%)	25% (16% to 38%)	3	147	0%, 22.5%^a^	5%, 41%, 58%^a^
Hib	15	1,373	25% (4% to 41%)	25% (4% to 44%)	3	187	0%, 14%, 16%^a^	0%, 13%, 38%^a^
Meningococcus	8	1,065	4% (1% to 13%)	7% (3% to 21%)	2	306	0%^a^	3%, 9%^a^
All bacteria	27	4,245	27% (1% to 75%)	18% (4% to 57%)	10	1,118	0% (0% to 18%)	25% (3% to 47%)

^a^Actual values.

CFR = case fatality ratio; Hib = *Haemophilus influenzae *type b.

## Discussion

In this review, we obtained comprehensive, up to date information on the burden of sequelae associated with bacterial meningitis in African children. We included 37 articles with sequelae data from 1980 to 2008, while an earlier review based sequelae estimates only on 10 articles published between 1970 and 2000 [3]. We estimated that the median risk of in-hospital sequelae was 25% for pneumococcal meningitis, 25% for Hib meningitis and 7% for meningococcal meningitis, while the median risk of post-discharge sequelae was 25% for all pathogens combined. These estimates are slightly lower than those found in the earlier African literature review [3], but the higher risk of sequelae for pneumococcal and Hib meningitis compared to meningococcal meningitis is consistent across the two reviews. The median CFR estimates in our review were 35% and 25% for pneumococcal and Hib meningitis, respectively, and thus slightly lower than the findings from two previous reviews [3,9].

Few studies report on the nature of the neuropsychological deficit beyond vision loss, hearing loss, seizures and gross motor impairment. Our database included more detailed and subtle neuropsychological deficit categories in keeping with the World Health Organization (WHO) international classification of functioning, disability and health [7]. This highlighted gaps in knowledge for the population studied: particularly lacking are data on cognitive delay, speech/language problems and behavioural problems.

In the studies we included, children with meningitis were treated in hospital with antibiotics recommended by the WHO (third generation cephalosporins or ampicillin plus chloramphenicol), and in some cases steroids [18]. Our findings suggest that even with appropriate antibiotic treatment, up to half of all children affected by pneumococcal and Hib meningitis die or experience clinically evident sequelae prior to hospital discharge. Increasing resistance to penicillin and chloramphenicol among pneumococcal strains may contribute to worse outcomes in some cases where access to third generation cephalosporins is limited. In our review, 15 of the articles (41%) reported using third generation cephalosporins as a first-line antibiotic in at least some cases. There were no clear trends in in-hospital sequelae risk over time likely because of relatively little heterogeneity in first-line therapies during the time period studied. Updated WHO guidelines promote the use of ceftriaxone in epidemic and non-epidemic settings as the first-line therapy for bacterial meningitis in Africa [19].

Further, a small number of studies included in the review show an increased risk of death and a 25% risk of long-term disability subsequent to discharge in children hospitalised for bacterial meningitis. The median estimates for in-hospital and post-discharge sequelae in our review did not differ greatly even though some sequelae following bacterial meningitis are expected to resolve over time [20]. African children experience the highest incidence rates for pneumococcal and Hib meningitis globally [9]. The high incidence, case fatality and sequelae risk due to bacterial meningitis underscore the public health importance of this disease and its poor outcome among children in Africa.

The articles included in this review represent 21 of the 52 African countries. In all, 44% of children with in-hospital outcome data were from the western Africa meningitis belt region and 72% of those with post-discharge data were from the northern Africa region. There were no major regional differences for study estimates of in-hospital sequelae risk associated with each of the three main bacteria (Table 5). The post-discharge sequelae studies had very few subjects overall and represent only seven countries. Among the post-discharge studies, the two northern Africa studies found the lowest risk of sequelae associated with individual bacterial pathogens (Table 7).

Our strict case definition for confirmed bacterial meningitis led to the exclusion of 42 articles with data on neuropsychological sequelae (49% of 86 studies). This definition was selected to ensure that cases of viral meningitis, which are generally less severe, were excluded and to enable us to calculate pathogen-specific estimates for sequelae and mortality. However, this methodological choice limits the generalisibility of our findings, given the small number and regional provenance of the remaining studies.

Many African children have limited access to medical care. Our review includes only patients who were admitted to a hospital, of which 77% were tertiary referral or teaching hospitals, had diagnostic testing for bacterial meningitis and received appropriate antibiotic therapy. These children are likely to have improved outcomes compared to children treated in lower-level facilities, where antibiotics are not always available, and children who did not reach medical care. Thus, our review likely underestimates the overall burden of sequelae and mortality in African children with bacterial meningitis.

This review identified very few studies with data on post-discharge outcomes of bacterial meningitis among African children. High loss to follow-up in these studies may lead to biased results, while small sample sizes limit the precision of our findings. However, a post-discharge study of children with pneumococcal meningitis from Bangladesh found that 11% died and 49% had long-term sequelae detectable 12 to 24 months post infection [21]. This study had a low loss to follow-up rate of 11%, and the proportion of children affected was on par with a similar study from The Gambia [22]. A recent review of the global burden of meningitis sequelae was conducted and found the highest risk of long-term sequelae in Africa compared to all other regions (Karen Edmond, senior lecturer in infectious disease epidemiology, London School of Hygiene and Tropical Medicine, personal communication). Limited access to health care and poor health indicators make many African children highly susceptible to adverse outcomes following bacterial meningitis.

Further, the social and economic burden of survivors with neuropsychological sequelae is little studied in Africa. Most studies included in this review diagnosed sequelae based on clinical exam alone and thus represent profound deficits that would severely affect a child's developmental potential. In the absence of follow-up specialty services for affected children, major sequelae can result in lifelong disability. This in turn affects the disabled child's entire family, as caregivers must choose between caring for the disabled child, providing for siblings and working outside the home to supplement the family's income [21]. In a South African study following children with TB meningitis and their families, 35% of previously-employed mothers had to stop working and one in five families experienced a financial loss as a result of the child's illness; half of the schoolgoing children failed at least one school grade [23]. The societal burden of hearing loss is best documented, and in several countries meningitis has been consistently found to be a common cause of profound hearing loss [24-26].

Our review provides no information on the effect of HIV on meningitis outcomes. HIV may shift the relative frequencies of bacterial pathogens causing meningitis and contribute to childhood malnutrition, which was associated with worse prognosis in our risk factor analysis. A study from Malawi found a higher rate of mortality and recurrent disease in HIV-infected children with bacterial meningitis [27]. Another study from South Africa found a higher rate of pneumococcal meningitis and poor outcomes in HIV-infected children [28]. More research is needed on the effects of the HIV pandemic on bacterial meningitis outcomes, particularly in the high HIV prevalence areas of middle, southern and eastern Africa.

The three main bacterial causes of postneonatal childhood meningitis are all vaccine preventable. Even with appropriate treatment, pneumococcal and Hib meningitis kill approximately one third of affected children and cause clinically evident sequelae in a quarter of survivors prior to hospital discharge. Hib conjugate vaccine use has nearly eliminated Hib meningitis in many African countries [29,30]. In Uganda, it was estimated that the vaccine prevents 1,000 cases of severe meningitis sequelae each year in the country's population of 5.3 million children under 5 years of age [29]. Pneumococcal conjugate vaccines (PCVs) are effective against invasive pneumococcal disease in African children, including meningitis [31]. In light of the high incidence of pneumococcal disease and its poor outcomes, these vaccines could prevent substantial numbers of meningitis-associated disabilities and deaths. PCV introduction into GAVI-eligible countries is underway and subsequent health and economic benefits will be closely monitored. Finally, though mortality and sequelae risk following meningococcal meningitis are not as high as with pneumococcal or Hib meningitis, the absolute numbers of sequelae and deaths occurring during meningococcal meningitis epidemics are tremendous. The epidemic potential of meningococcal meningitis has focused much attention on the introduction of meningococcal polysaccharide and conjugate vaccines in countries of the African meningitis belt. In addition, the related clinical syndrome of meningococcal sepsis, a fulminant disease that has been associated with CFRs of up to 80%, could decrease with the expanded use of meningococcal vaccine [32].

Evaluations of the health impact and cost effectiveness of conjugate vaccines should incorporate the economic and social impact of meningitis morbidity and mortality on families, many of which have little financial reserve to care for a child with a long-term disability. This review mainly includes children treated at tertiary care facilities and therefore underestimates the sequelae and mortality risk associated with bacterial meningitis. Thus, the true cost of meningitis and the true benefit of bacterial conjugate vaccines are likely to be tremendous.

## Conclusion

Bacterial meningitis is a serious infection that has high risk of sequelae as well as mortality in African children. Even with appropriate treatment, pneumococcal and Hib meningitis kill approximately one third of affected children and cause clinically evident sequelae in a quarter of survivors prior to hospital discharge. The three leading causes of bacterial meningitis in childhood are vaccine preventable, and the regular use of these conjugate vaccines would reduce the high burden of morbidity and mortality in both epidemic and endemic settings.

## Competing interests

The authors declare that they have no competing interests.

## Authors' contributions

MR managed the review team, screened citations for inclusion or exclusion, reviewed the data analysis, drafted and revised the manuscript. AU screened citations, helped manage the review team, reviewed the data analysis and helped draft the manuscript. LS designed, managed and cleaned the database, conducted the data analysis and created the results tables. JM contributed to study design, oversaw database management and analysis and edited the manuscript. FW provided technical input to the process. OL conceived of the project and provided technical oversight to design and execute the review. All authors read and approved the final manuscript.

## Pre-publication history

The pre-publication history for this paper can be accessed here:



## Supplementary Material

Additional File 1 **Literature search strategy**. Keywords, search terms and dates of execution for literature search in each of the eight databases used. Click here for file
