# Supplementary material for: Sequelae due to bacterial meningitis among African children: a systematic literature review
Source: BMC Med. 2009 Sep 14;7:47. doi: 10.1186/1741-7015-7-47 (PMC2759956; doi:10.1186/1741-7015-7-47)
Supplement: Additional File 1 — Literature search strategy. Keywords, search terms and dates of execution for literature search in each of the eight databases used. [file 1741-7015-7-47-S1.doc]

# Sequelae due to bacterial meningitis among African children: a systematic literature review

Authors: Meenakshi Ramakrishnan, Aaron J. Ulland, Laura C. Steinhardt, Jennifer C. Moïsi, Fred Were and Orin S. Levine

# Additional file 1: Literature search strategy

# PubMed/Medline—513 results

# Performed: June 21, 2008

# "Meningitis, Haemophilus"[Mesh] OR "Meningitis, Pneumococcal"[Mesh] OR Pneumococcal meningitis[tiab] OR haemophilus meningitis[tiab] OR Neisseria meningitis[tiab] OR Neisseria meningitides[tiab] OR bacterial meningitis[tiab] OR "meningitis, bacterial"[MeSH Terms] OR "meningitis, meningococcal"[MeSH Terms] OR Meningococcal Meningitis[tiab] OR "meningococcal vaccines"[MeSH Terms] OR "meningococcal vaccines"[tiab] OR "meningococcal vaccine"[tiab]

# AND

# infant[mesh] OR infant* OR baby OR child[mesh] OR child* OR children OR teen* OR youth* OR adolescent[mesh] OR adolesc*

# AND

# africa [Mesh] OR africa [tiab] OR algeria [tiab] OR angola [tiab] OR benin [tiab] OR botswana [tiab] OR burkina faso [tiab] OR burundi [tiab] OR cameroon [tiab] OR cape verde [tiab] OR central african republic [tiab] OR chad [tiab] OR comoros [tiab] OR democratic republic of the congo [tiab] OR republic of the congo [tiab] OR congo-brazzaville [tiab] OR cote d'ivoire OR ivory coast [tiab] OR djibouti [tiab] OR egypt [tiab] OR equatorial guinea [tiab] OR eritrea [tiab] OR ethiopia [tiab] OR gabon [tiab] OR gambia [tiab] OR ghana [tiab] OR guinea [tiab] OR guinea-bissau [tiab] OR kenya [tiab] OR lesotho [tiab] OR libya [tiab] OR madagascar [tiab] OR malawi [tiab] OR mali [tiab] OR mauritania [tiab] OR mauritius [tiab] OR mayotte [tiab] OR morocco [tiab] OR mozambique [tiab] OR namibia [tiab] OR niger [tiab] OR nigeria [tiab] OR rwanda [tiab] OR senegal [tiab] OR seychelles [tiab] OR sierra leone [tiab] OR somalia [tiab] OR south africa [tiab] OR sudan [tiab] OR swaziland [tiab] OR tanzania [tiab] OR togo [tiab] OR tunisia [tiab] OR uganda [tiab] OR western sahara [tiab] OR zambia [tiab] OR zimbabwe [tiab] OR algeria [MeSH] OR angola [MeSH] OR benin [MeSH] OR botswana [MeSH] OR burkina faso [MeSH] OR burundi [MeSH] OR cameroon [MeSH] OR cape verde [MeSH] OR central african republic [MeSH] OR chad [MeSH] OR comoros [MeSH] OR democratic republic of the congo [MeSH] OR republic of the congo [MeSH] OR congo-brazzaville [MeSH] OR cote d'ivoire OR ivory coast [MeSH] OR djibouti [MeSH] OR egypt [MeSH] OR equatorial guinea [MeSH] OR eritrea [MeSH] OR ethiopia [MeSH] OR gabon [MeSH] OR gambia [MeSH] OR ghana [MeSH] OR guinea [MeSH] OR guinea-bissau [MeSH] OR kenya [MeSH] OR lesotho [MeSH] OR libya [MeSH] OR madagascar [MeSH] OR malawi [MeSH] OR mali [MeSH] OR mauritania [MeSH] OR mauritius [MeSH] OR mayotte [MeSH] OR morocco [MeSH] OR mozambique [MeSH] OR namibia [MeSH] OR niger [MeSH] OR nigeria [MeSH] OR rwanda [MeSH] OR senegal [MeSH] OR seychelles [MeSH] OR sierra leone [MeSH] OR somalia [MeSH] OR south africa [MeSH] OR sudan [MeSH] OR swaziland [MeSH] OR tanzania [MeSH] OR togo [MeSH] OR tunisia [MeSH] OR uganda [MeSH] OR western sahara [MeSH] OR zambia [MeSH] OR zimbabwe [MeSH]

# Embase—599 results

# Performed: June 22, 2008

# 'meningitis, pneumococcal'/exp OR 'meningitis, pneumococcal' OR 'pneumococcal meningitis'/exp OR 'pneumococcal meningitis' OR 'haemophilus meningitis' OR 'neisseria meningitis' OR 'neisseria meningitides'/exp OR 'neisseria meningitides' OR 'bacterial meningitis'/exp OR 'bacterial meningitis' OR 'meningitis, bacterial'/exp OR 'meningitis, bacterial' OR 'meningitis, meningococcal'/exp OR 'meningitis, meningococcal' OR 'meningococcal meningitis'/exp OR 'meningococcal meningitis' OR 'meningococcal vaccines'/exp OR 'meningococcal vaccines' OR 'meningococcal vaccine'/exp OR 'meningococcal vaccine'

# AND

# 'child'/exp OR 'adolescent'/exp OR child OR infant OR baby OR teen OR youth OR teens OR teenager OR teenagers OR children OR infants OR babies OR youths OR adolescent OR adolescents OR adolescence

# AND

# 'africa'/exp OR 'africa'/exp OR 'africa' OR 'algeria'/exp OR 'algeria' OR 'angola'/exp OR 'angola' OR 'benin'/exp OR 'benin' OR 'botswana'/exp OR 'botswana' OR 'burkina faso'/exp OR 'burkina faso' OR 'burundi'/exp OR 'burundi' OR 'cameroon'/exp OR 'cameroon' OR 'cape verde'/exp OR 'cape verde' OR 'central african republic'/exp OR 'central african republic' OR 'chad'/exp OR 'chad' OR 'comoros'/exp OR 'comoros' OR 'democratic republic of the congo'/exp OR 'democratic republic of the congo' OR 'congo'/exp OR 'congo' OR 'republic of the congo' OR 'congo brazzaville'/exp OR 'congo brazzaville' OR 'ivory coast'/exp OR 'ivory coast' OR 'djibouti'/exp OR 'djibouti' OR 'egypt'/exp OR 'egypt' OR 'equatorial guinea'/exp OR 'equatorial guinea' OR 'eritrea'/exp OR 'eritrea' OR 'ethiopia'/exp OR 'ethiopia' OR 'gabon'/exp OR 'gabon' OR 'gambia'/exp OR 'gambia' OR 'ghana'/exp OR 'ghana' OR 'guinea'/exp OR 'guinea' OR 'guinea bissau'/exp OR 'guinea bissau' OR 'kenya'/exp OR 'kenya' OR 'lesotho'/exp OR 'lesotho' OR 'libya'/exp OR 'libya' OR 'madagascar'/exp OR 'madagascar' OR 'malawi'/exp OR 'malawi' OR 'mali'/exp OR 'mali' OR 'mauritania'/exp OR 'mauritania' OR 'mauritius'/exp OR 'mauritius' OR 'mayotte'/exp OR 'mayotte' OR 'morocco'/exp OR 'morocco' OR 'mozambique'/exp OR 'mozambique' OR 'namibia'/exp OR 'namibia' OR 'niger'/exp OR 'niger' OR 'nigeria'/exp OR 'nigeria' OR 'rwanda'/exp OR 'rwanda' OR 'senegal'/exp OR 'senegal' OR 'seychelles'/exp OR 'seychelles' OR 'sierra leone'/exp OR 'sierra leone' OR 'somalia'/exp OR 'somalia' OR 'south africa'/exp OR 'sudan'/exp OR 'sudan' OR 'swaziland'/exp OR 'swaziland' OR 'tanzania'/exp OR 'tanzania' OR 'togo'/exp OR 'togo' OR 'tunisia'/exp OR 'tunisia' OR 'uganda'/exp OR 'uganda' OR 'zambia'/exp OR 'zambia' OR 'zimbabwe'/exp OR 'zimbabwe'

# Pascal BioMed—216 results

# Performed: June 22, 2008

((( Africa )or ( algeria )or( angola )or( benin )or( botswana )or( burkina faso )or( burundi )or( cameroon )or( cape verde )or( central african republic )or( chad )or( comoros )or( democratic republic of the congo )or( republic of the congo )or( congo-brazzaville )or( cote d'ivoire )or( ivory coast )or( djibouti )or( egypt )or( equatorial guinea )or( eritrea )or( ethiopia )or( gabon )or( gambia )or( ghana )or( guinea )or( guinea-bissau )or( kenya )or( lesotho )or( libya )or( madagascar )or( malawi )or( mali )or( mauritania )or( mauritius )or( mayotte )or( morocco )or( mozambique )or( namibia )or( niger )or( nigeria )or( rwanda )or( senegal )or( seychelles )or( sierra leone )or( somalia )or( south africa )or( sudan )or( swaziland )or( tanzania )or( togo )or( tunisia )or( uganda )or( western sahara )or( zambia )or( zimbabwe ))

and

((( Haemophilus )or( Pneumococcal )or( Neisseria )or( bacterial )or( meningococcal )or( Hemophilus )or( Pneumococal )or( Neisseria )or( meningococcal ))

and

(( meningitis )or( meningitides ))))

and

(( infant )or( infants )or( baby )or( babies )or( child )or( children )or( teen )or( teens )or( teenager )or( teenagers )or( youth )or( youths )or( adolescent )or( adolescents )or(adolescence ))

**Global Health**—365 results

Performed: June 24, 2008

1 (adolesc$ or teen$ or youth or children or infant$ or child$ or baby or babies).mp. [mp=abstract, title, original title, broad terms, heading words] 169641

2 exp bacterial meningitis/ or exp neisseria meningitidis/ 4497

3 haemophilus/ 4172

4 exp meningitis/ 5785

5 3 and 4 561

6 bacteremia.mp. or exp bacteraemia/ 4713

7 exp streptococcus/ 12429

8 4 and 7 940

9 ("haemophilus meningitis" or "Hemophilus Meningitis OR Streptococcus meningitis" or "meningococcal meningitis" or "Pneumococcal Meningitis").mp. [mp=abstract, title, original title, broad terms, heading words] 625

10 2 or 5 or 6 or 8 or 9 9287

11 exp "Great Lakes (Africa)"/ or exp Southern Africa/ or exp Central Africa/ or exp North Africa/ or exp Portuguese Speaking Africa/ or exp tropical Africa/ or exp Anglophone Africa/ or africa.mp. or exp "North-West (South Africa)"/ or exp "Africa South of Sahara"/ or exp East Africa/ or exp Africa/ or exp West Africa/ or exp South Africa/ or exp Francophone Africa/ 80302

12 (algeria or angola or benin or botswana or burkina faso or burundi or cameroon or cape verde or central african republic or chad or comoros or democratic republic of the congo or republic of the congo or congo-brazzaville or ivory coast or djibouti or egypt or equatorial guinea or eritrea or ethiopia or gabon or gambia or ghana or guinea or guinea-bissau or kenya or lesotho or libya or madagascar or malawi or mali or mauritania or mauritius or mayotte or morocco or mozambique or namibia or niger or nigeria or rwanda or senegal or seychelles or sierra leone or somalia or south africa or sudan or swaziland or tanzania or togo or tunisia or uganda or western sahara or zambia or zimbabwe).mp. [mp=abstract, title, original title, broad terms, heading words] 82683

13 11 or 12 95224

14 exp children/ or exp adolescents/ or exp infants/ 120033

15 1 or 14 169641

**Cochrane Search**—131 results

Performed: August 12, 2008

ID Search Hits Edit Delete

#1 (africa OR algeria OR angola OR benin OR botswana OR "burkina faso" OR burundi OR cameroon OR "cape verde" OR "central african republic" OR chad OR comoros OR "democratic republic of the congo" OR "republic of the congo" OR "congo-brazzaville" OR "cote d'ivoire" OR "ivory coast" OR djibouti OR egypt OR "equatorial guinea" OR eritrea OR ethiopia OR gabon OR gambia OR ghana OR guinea OR "guinea-bissau" OR kenya OR lesotho OR libya OR madagascar OR malawi OR mali OR mauritania OR mauritius OR mayotte OR morocco OR mozambique OR namibia OR niger OR nigeria OR rwanda OR senegal OR seychelles OR "sierra leone" OR somalia OR "south africa" OR sudan OR swaziland OR tanzania OR togo OR tunisia OR uganda OR "western sahara" OR zambia OR zimbabwe) 7034 edit delete

#2 "bacterial meninigitis" or bacteremia or "streptococcus pneumoniae" or "haemophilus influenzae" or "neisseria meningitides" 2558 edit delete

#3 (adolescent or adolescents or teen or teens or youth or children or infant or infants or child or baby or babies) 110614 edit delete

#4 MeSH descriptor Child explode all trees 0 edit delete

#5 MeSH descriptor Adolescent explode all trees 58374 edit delete

#6 MeSH descriptor Infant explode all trees 9980 edit delete

#7 MeSH descriptor Meningitis, Haemophilus explode all trees 66 edit delete

#8 MeSH descriptor Meningitis, Pneumococcal explode all trees 56 edit delete

#9 MeSH descriptor Meningitis, Bacterial explode all trees 274 edit delete

#10 MeSH descriptor Meningitis, Meningococcal explode all trees 99 edit delete

#11 MeSH descriptor Meningococcal Vaccines explode all trees 164 edit delete

#12 (#3 OR #4 OR #5 OR #6) 110614 edit delete

#13 (#2 OR #7 OR #8 OR #9 OR #10 OR #11) 2838 edit delete

#14 (#1 AND #12 AND #13) 133 edit delete

**Web of Science**—76 results

Performed: June 30, 2008

Africa OR algeria OR angola OR benin OR botswana OR "burkina faso" OR burundi OR cameroon OR "cape verde" OR "central african republic" OR chad OR comoros OR "democratic republic of the congo" OR "republic of the congo" OR congo-brazzaville OR "cote d'ivoire" OR "ivory coast" OR djibouti OR egypt OR "equatorial guinea" OR eritrea OR ethiopia OR gabon

OR gambia OR ghana OR guinea OR guinea-bissau

AND

<http://apps.isiknowledge.com/summary.do?product=WOS&doc=1&qid=4&SID=2B8dJBIdccOKihpM5Fm&search_mode=GeneralSearch>

Topic=(meningitis OR meningitides) AND Topic=(Haemophilus OR Pneumococcal OR Neisseria OR bacterial OR meningococcal OR Hemophilus OR Pneumococal OR Neisseria OR meningococcal) AND Topic=(infant* OR bab* OR child* teen* OR youth* OR adolescen*)

OR

kenya OR lesotho OR libya OR madagascar OR malawi OR mali OR mauritania OR mauritius OR mayotte OR morocco OR mozambique OR namibia OR niger OR nigeria OR rwanda OR senegal OR seychelles OR "sierra leone" OR somalia OR "south africa" OR sudan OR swaziland OR Tanzania OR togo or tunisia or uganda or western sahara or zambia or zimbabwe

AND

<http://apps.isiknowledge.com/summary.do?product=WOS&doc=1&qid=4&SID=2B8dJBIdccOKihpM5Fm&search_mode=GeneralSearch>

Topic=(meningitis OR meningitides) AND Topic=(Haemophilus OR Pneumococcal OR Neisseria OR bacterial OR meningococcal OR Hemophilus OR Pneumococal OR Neisseria OR meningococcal) AND Topic=(infant* OR bab* OR child* teen* OR youth* OR adolescen*)

**Biological Abstracts (Biosis)—**120 results

Performed: June 26, 2008

(( infant )or( infants )or( baby )or( babies )or( child )or( children )or( teen )or( teens )or( teenager )or( teenagers )or( youth )or( youths )or( adolescent )or( adolescents )or(adolescence ))

and

(((MENINGITIS) or (MENINGITIS-) or (MENINGITIS-ASSOCIATED-CENTRAL-NERVOUS-SYSTEM-COMPLICATION-ROLE) or (MENINGITIS-ASSOCIATED-CENTRAL-NERVOUS-SYSTEM-COMPLICATIONS) or (MENINGITIS-ASSOCIATED-HEARING-LOSS))

and

("Bacterial-Disease" in CB,CC,DS,GE,MC,MQ,OR,PS,SD,ST,TM,TN)) or ((Haemophilus or Pneumococcal or Hemophilus or Pneumococal or Neisseria or meningococcal)

AND

("Africa-" in CB,CC,DS,GE,MC,MQ,OR,PS,SD,ST,TM,TN) or ("Algeria-" in CB,CC,DS,GE,MC,MQ,OR,PS,SD,ST,TM,TN) or ("Angola-" in CB,CC,DS,GE,MC,MQ,OR,PS,SD,ST,TM,TN) or ("Benin-" in CB,CC,DS,GE,MC,MQ,OR,PS,SD,ST,TM,TN) or ("Botswana-" in CB,CC,DS,GE,MC,MQ,OR,PS,SD,ST,TM,TN) or ("Burkina-Faso" in CB,CC,DS,GE,MC,MQ,OR,PS,SD,ST,TM,TN) or ("Burundi-" in CB,CC,DS,GE,MC,MQ,OR,PS,SD,ST,TM,TN) or ("Cameroon-" in CB,CC,DS,GE,MC,MQ,OR,PS,SD,ST,TM,TN) or ("Central-African-Republic" in CB,CC,DS,GE,MC,MQ,OR,PS,SD,ST,TM,TN) or ("Chad-" in CB,CC,DS,GE,MC,MQ,OR,PS,SD,ST,TM,TN) or ("Congo-" in CB,CC,DS,GE,MC,MQ,OR,PS,SD,ST,TM,TN) or ("Congo-River" in CB,CC,DS,GE,MC,MQ,OR,PS,SD,ST,TM,TN) or ("Djibouti-" in CB,CC,DS,GE,MC,MQ,OR,PS,SD,ST,TM,TN) or ("Egypt-" in CB,CC,DS,GE,MC,MQ,OR,PS,SD,ST,TM,TN) or ("Equatorial-Guinea" in CB,CC,DS,GE,MC,MQ,OR,PS,SD,ST,TM,TN) or ("Eritrea-" in CB,CC,DS,GE,MC,MQ,OR,PS,SD,ST,TM,TN) or ("Ethiopia-" in CB,CC,DS,GE,MC,MQ,OR,PS,SD,ST,TM,TN) or ("Gabon-" in CB,CC,DS,GE,MC,MQ,OR,PS,SD,ST,TM,TN) or ("Gambia-" in CB,CC,DS,GE,MC,MQ,OR,PS,SD,ST,TM,TN) or ("Ghana-" in CB,CC,DS,GE,MC,MQ,OR,PS,SD,ST,TM,TN) or ("Guinea-" in CB,CC,DS,GE,MC,MQ,OR,PS,SD,ST,TM,TN) or ("Guinea-Bissau" in CB,CC,DS,GE,MC,MQ,OR,PS,SD,ST,TM,TN) or ("Ivory-Coast" in CB,CC,DS,GE,MC,MQ,OR,PS,SD,ST,TM,TN) or ("Lesotho-" in CB,CC,DS,GE,MC,MQ,OR,PS,SD,ST,TM,TN) or ("Liberia-" in CB,CC,DS,GE,MC,MQ,OR,PS,SD,ST,TM,TN) or ("Libya-" in CB,CC,DS,GE,MC,MQ,OR,PS,SD,ST,TM,TN) or ("Madagascar-" in CB,CC,DS,GE,MC,MQ,OR,PS,SD,ST,TM,TN) or ("Malawi-" in CB,CC,DS,GE,MC,MQ,OR,PS,SD,ST,TM,TN) or ("Mali-" in CB,CC,DS,GE,MC,MQ,OR,PS,SD,ST,TM,TN) or ("Mauritania-" in CB,CC,DS,GE,MC,MQ,OR,PS,SD,ST,TM,TN) or ("Morocco-" in CB,CC,DS,GE,MC,MQ,OR,PS,SD,ST,TM,TN) or ("Mozambique-" in CB,CC,DS,GE,MC,MQ,OR,PS,SD,ST,TM,TN) or ("Namibia-" in CB,CC,DS,GE,MC,MQ,OR,PS,SD,ST,TM,TN) or ("Niger-" in CB,CC,DS,GE,MC,MQ,OR,PS,SD,ST,TM,TN) or ("Nigeria-" in CB,CC,DS,GE,MC,MQ,OR,PS,SD,ST,TM,TN) or ("Rwanda-" in CB,CC,DS,GE,MC,MQ,OR,PS,SD,ST,TM,TN) or ("Sao-Tome-And-Principe" in CB,CC,DS,GE,MC,MQ,OR,PS,SD,ST,TM,TN) or ("Senegal-" in CB,CC,DS,GE,MC,MQ,OR,PS,SD,ST,TM,TN) or ("Sierra-Leone" in CB,CC,DS,GE,MC,MQ,OR,PS,SD,ST,TM,TN) or ("Somalia-" in CB,CC,DS,GE,MC,MQ,OR,PS,SD,ST,TM,TN) or ("South-Africa" in CB,CC,DS,GE,MC,MQ,OR,PS,SD,ST,TM,TN) or ("Sudan-" in CB,CC,DS,GE,MC,MQ,OR,PS,SD,ST,TM,TN) or ("Swaziland-" in CB,CC,DS,GE,MC,MQ,OR,PS,SD,ST,TM,TN) or ("Tanzania-" in CB,CC,DS,GE,MC,MQ,OR,PS,SD,ST,TM,TN) or ("Togo-" in CB,CC,DS,GE,MC,MQ,OR,PS,SD,ST,TM,TN) or ("Tunisia-" in CB,CC,DS,GE,MC,MQ,OR,PS,SD,ST,TM,TN) or ("Uganda-" in CB,CC,DS,GE,MC,MQ,OR,PS,SD,ST,TM,TN) or ("Zaire-" in CB,CC,DS,GE,MC,MQ,OR,PS,SD,ST,TM,TN) or ("Zambia-" in CB,CC,DS,GE,MC,MQ,OR,PS,SD,ST,TM,TN) or ("Zimbabwe-" in CB,CC,DS,GE,MC,MQ,OR,PS,SD,ST,TM,TN)

Additional search with French terms, no additional hits:

(pediatriques or pediatrie or enfant or enfants)

and

(meningites or meningite)

and

(bacteriologiques or bacterienne or bacteriennes or pneumocoque or meningocoque)

**African Index Medicus** -- 18 results

Performed: July 4, 2008

pédiatriques OR pédiatrie OR enfant OR enfants OR infant OR infants OR child OR children OR baby OR babies OR adolescent OR adolescents OR adolescence OR teen OR teens OR teenagers [Palavras]

and

bactériologiques OR bactérienne OR bactériennes OR Haemophilus OR Pneumococcal OR Neisseria OR bacterial OR meningococcal OR Hemophilus OR Pneumococal OR Neisseria OR meningococcal [Palavras]

and

méningites OR méningite OR meningitis OR meningitides [Palavras]
